# Inventions and New Things

**Published:** 1890-04

**Authors:** J. Taft


### Inventions and New Things.

BY J. TAFT.

In the early periods of the practice of dentistry the instruments, appliances and modes, were exceedingly simple and elementary, compared with those in use now. The history of this progress and development is very interesting to those who care to make it a subject of investigation and study.

There is a present practical side to this subject of the introduction of new modes, appliances and materials, that is of special interest to every practitioner, and reference is not here especially made to the intrinsic value of these things, but to the mode of introduction and use by the profession. The usual course when a new invention is made is to secure a patent. Two motives impel to this course; the one by far the most prominent and frequent is that of pecuniary profit—indeed, this is almost universal—the other is to establish and maintain the priority of invention, and whatever of credit may attach to that fact. Against neither of these motives can aught be said when in reasonable exercise. Usually by patents an inventor can secure to himself the priority of discovery or invention; but the cases in which an actual inventor receives any considerable money profit, are rare indeed. Patents for valuable inventions generally fall into the hands of others, who have more money and greater business talent than the inventor.

So far as patents pertaining to dentistry are concerned, by far the larger proportion have proved utterly worthless to the inventors or any one else; some have been good but never developed; some have been of real worth, more or less pronounced; some of these have been used in a way that was proper and right, speaking in a commercial sense (leaving the professional aspect out of view altogether). Others have been used in ways that were regarded as oppressive and unjust—extortionate; such for example as the rubber patents of twenty-five years ago, under the Bacon administration. Patents are now being used in a manner that seem to the profession to be oppressive and unjust—it is needless here to specify—but this opinion prevails in the profession almost universally. Out of this condition of things has arisen an organization the object of which is self-protection, not only from monopolies that are now in operation but from any that may in the future spring up. This organization is known as the "Dental Protective Association of the United States." Doubtless almost every dentist in the country knows something of this effort, and a large proportion have been fully informed on the subject, and quite a goodly number have allied themselves with it. This association arose out of a necessity; affairs had come to the point where it was either combined resistance or submission, to what was regarded as a merciless monopoly. As the entire profession was, is, or may be affected by the operations of the

International Tooth Crown Company, it is very natural that they are or should be as interested in an effort for their own protection; and that this interest has already assumed large proportions and is rapidly on the increase, there is abundant evidence. At a meeting of over one hundred dentists in the City of New York the other day, the following resolution was unanimously adopted:

*Resolved*, That we thoroughly endorse the Dental Protective Association of the United States, and urge upon every member of the dental profession to join the association, and send to Dr. J. N. Crouse, of Chicago, its President, the initiation fee of ten dollars. WM JARVIE, Secretary.

And in addition to this every member of that meeting became a member of the Protective Association, and contributed his mite to its resources.

This is an illustration of the feeling that is abroad throughout the profession in regard to this matter.

In this movement there is manifest a prophecy of better things for the future.

The evils which called forth this association have existed for a long time, but such was the apathy in the profession, in regard to the subject, that it has assumed proportions that are alarming; and which, without arrest, will become a burden and a great oppression.

The entire subject of patents, nostrums and secret remedies could with great propriety, and indeed should receive the special attention of the Protective Association, and thereby the profession would receive great benefit.

To relieve the profession, to some extent, from the embarrassment that attaches to the introduction of new inventions, appliances and instruments and modes, as now in vogue, it would seem practicable to organize, either through the Dental Protective Association, or the American Dental Association, or both jointly, a Commission, consisting of three or more experts, who should examine and decide upon the merits of every new invention or improvement, whether pertaining to instruments, appliances, materials or processes, and secret preparations as well, whether patented or not. Such a commission should, of course, be thoroughly competent, free from prejudice and bias of every sort, one whose opinions and decisions would not only have the respect, but command the confidence of the profession in the matters committed to its charge.

Such a course would afford protection to the profession, not only in regard to veritable frauds, but as to many things that are in themselves worthless, but are not presented and pressed in a fraudulent way. It may be that there are some things with which such a course would not be practicable; they would, however, be very few, and possibly none would be found, but it would be entirely practicable with the very large proportion of new things.

In regard to some things it may be said, that much time is required to determine their value or merits; while this is true, no more time would be required for determination by such a commission than by the individual members of the profession, and besides such examiners would have more experience and far more extensive facilities for making thorough tests. And further than this it may be said that now and again a person will be found who can make a success with that which to most persons proves on trial to be worthless; such exceptional cases should not in any sense be a guide for all others.

There are some who always prefer to decide for themselves. This privilege would in no wise be abridged by the scheme here suggested; but the large proportion of the profession would gladly avail themselves of the decision of such a commission, and would be vastly profited thereby. Such a course would be the means of weeding out all worthless things that might be pressed forward solely because they were new, and would greatly enhance the value, success and usefulness of the really meritorious.

This method could be further expanded, so as to embrace a reasonable compensation to all who should bring forth, or invent any thing new and really meritorious. And thus all the really valuable inventions, improvements, etc., would become available to the profession without being clogged with patent fees, royalties, etc. Such an arrangement would be more satisfactory to the inventors than the present, and doubtless more beneficial to the profession; for then every one would have some assurance as to the value of any new device or process before adopting it in practice; and money would not be wasted on worthless things. It would also prevent the fruitless waste of money in embarrassing litigation.

Such a commission, if properly organized and sustained, would be of incalculable benefit to the profession; it would wholly revolutionize the method of the introduction to the profession of inventions, improvements, processes and preparations, and afford a protection and a guarantee that could in no other way be secured.

Such an arrangement would bring the scientific, practical and financial interests of the profession into closer relationship than has hitherto existed, or than could be effected in any other way, so far as now appears; and not only this, but it would serve as a strong bond of union between the members of the profession; and every thing that tends in this direction should be appreciated and fostered.

If the profession, or a majority of its members, should become members of the Protective Association, and an organization such as is here suggested should be effected, and its work properly carried out, it would be a death blow to dental patents and nostrums.

The aim of this paper is to give some preliminary suggestions and thoughts that may stimulate others to think in the same direction, and prepare for progress in the future when the way shall seem to be open.

---

INDIA INK, stated to be identical with the genuine, is prepared by Piffard (*Chem. News*), by treating excess of champhor with concentrated sulphuric acid for twenty-four hours. The resultant gelatinous, reddish mass is then thoroughly heated to drive off the excess of acid and camphor. A black pigment remains which seems to dissolve in water, and produces a permanent, reliable ink.

